# Preserved Contextual Cueing in Realistic Scenes in Patients with Age-Related Macular Degeneration

**DOI:** 10.3390/brainsci10120941

**Published:** 2020-12-07

**Authors:** Stefan Pollmann, Lisa Rosenblum, Stefanie Linnhoff, Eleonora Porracin, Franziska Geringswald, Anne Herbik, Katja Renner, Michael B. Hoffmann

**Affiliations:** 1 Department of Experimental Psychology, Otto-von-Guericke-University, Postfach 4120, 39016 Magdeburg, Germany; erosenblum94@gmail.com (L.R.); stefanie.linnhoff@med.ovgu.de (S.L.); eleonora.porracin@gmail.com (E.P.); franziska.geringswald@gmail.com (F.G.); 2 Center for Behavioral Brain Sciences, Otto-von-Guericke-University, 39016 Magdeburg, Germany; michael.hoffmann@med.ovgu.de; 3 Beijing Key Laboratory of Learning and Cognition and School of Psychology, Capital Normal University, Beijing 100048, China; 4 Laboratoire de Neurosciences Cognitives UMR 7291, Aix-Marseille Université & CNRS, 13331 Marseille, France; 5 Department of Ophthalmology, Otto-von-Guericke-University, 39016 Magdeburg, Germany; anne.herbik@ovgu.de; 6 Eye Clinic Am Johannisplatz, 04103 Leipzig, Germany; renner@augen-leipzig.de

**Keywords:** visual search, vision loss, incidental learning, macular degeneration, fovea

## Abstract

Foveal vision loss has been shown to reduce efficient visual search guidance due to contextual cueing by incidentally learned contexts. However, previous studies used artificial (T- among L-shape) search paradigms that prevent the memorization of a target in a semantically meaningful scene. Here, we investigated contextual cueing in real-life scenes that allow explicit memory of target locations in semantically rich scenes. In contrast to the contextual cueing deficits in artificial scenes, contextual cueing in patients with age-related macular degeneration (AMD) did not differ from age-matched normal-sighted controls. We discuss this in the context of visuospatial working-memory demands for which both eye movement control in the presence of central vision loss and memory-guided search may compete. Memory-guided search in semantically rich scenes may depend less on visuospatial working memory than search in abstract displays, potentially explaining intact contextual cueing in the former but not the latter. In a practical sense, our findings may indicate that patients with AMD are less deficient than expected after previous lab experiments. This shows the usefulness of realistic stimuli in experimental clinical research.

## 1. Introduction

When we enter an environment, our eye movements can be guided by memory of the same or similar environments that we encountered in the past. When you think of your kitchen, you can explicitly tell where the refrigerator is and in which cupboard you will find a coffee mug. Similarly, when participants were asked to find a target in a realistic scene, their response time was dramatically reduced when scenes—with the target placed at the same location—were repeated [1]. Unsurprisingly, participants were explicitly aware of the location of targets in the scenes when tested at the end of the experiment. However, search facilitation in repeated displays was also observed when the “scene” was a symbolic display, typically consisting of a T-shaped target among L-shaped distractors in a randomly generated configuration without any semantic meaning. Nevertheless, response time was reduced when these arbitrary distractor-target configurations were repeatedly presented. This contextual cueing effect, initially described by [2], is an incidental learning effect. It occurs even though participants are not told about the repetition of displays and often in the absence of explicit recognition of the repeated spatial configuration. Although the implicit nature of contextual cueing has been debated [3], it has been repeatedly observed that only a fraction of displays can be explicitly recognized [4,5]. Furthermore, the amount of search facilitation, i.e., the reduction in response times in repeated displays, is typically not correlated with the confidence of recognizing a repeated display [6]. Thus, contextual cueing in symbolic displays appears to be due to incidental learning processes that occur at least partially in the absence of explicit recognition, whereas contextual cueing in realistic scenes additionally draws on explicit memory.

In a previous study, we showed that incidental contextual cueing in symbolic displays was severely reduced in a group of patients with age-related macular degeneration (AMD) [7]. AMD is a degenerative disease leading to vision loss, particularly in the macula, typically more strongly in one eye. No contextual cueing was observed when patients were tested with their worse eye. When tested binocularly—as the more ecological viewing condition—search times were, overall, faster for repeated displays, but, in contrast to the control group, the improvement did not increase over time, as indicated by a non-significant configuration × epoch interaction [7]. The loss of contextual cueing after foveal vision loss could be confirmed in a study with normal-sighted young participants in whom a foveal scotoma was simulated with gaze-contingent display methods [8] as well as in a further study with gaze-contingent central scotoma simulation [9]. In the latter study, however, when the simulated central scotoma was removed after a training session, allowing normal viewing of the search displays, the response-time advantage for repeated displays (the same displays as in the central scotoma training phase) was immediately present. This pattern showed that search with a simulated central scotoma did not prevent the learning of repeated target-distractor patterns, but rather prevented the use of these memories for efficient search guidance. Once the scotoma simulation was removed, the configurations learned during the central scotoma search could be used to guide the search for the target when a display was repeated.

Thus, although incidental spatial configuration learning may occur in the presence of foveal vision loss, memory-guided search may suffer due to inefficient expression of learning. We know that the use of learned contexts for search guidance depends on visuospatial working memory. This has been shown by multiple studies using secondary working memory tasks to deplete working memory resources during search [10,11,12]; reviewed in [13]. These studies have shown that specifically the use of visuospatial working memory capacity by a secondary task reduced or abolished the search facilitation otherwise observed in repeated displays [11]. In the case of foveal vision loss, the much more top-down controlled exploration of the environment, characterized by fewer saccades, longer dwell times, and larger saccade amplitudes, puts high demands on visuospatial working memory, thereby preventing efficient search guidance by learned visuospatial contexts [9]. However, while the visuospatial working memory demands of top-down controlled search may interfere with holding learned visuospatial patterns in working memory for search guidance, this may be different for explicitly remembered target locations in realistic displays (e.g., “the cup on the kitchen table”). The latter can be retrieved and held available during search very efficiently by semantic memory templates [14,15], which do not compete with top-down controlled scene exploration for visuospatial working memory capacity. In support of this view, we recently reported that contextual cueing was intact for search in realistic displays with simulated gaze-contingent central or peripheral scotomata [16].

Therefore, in the present study, we hypothesized that the search in realistic scenes might enable patients with AMD to show contextual cueing scores at a level comparable to those of age-matched controls with intact (best corrected) vision.

## 2. Materials and Methods

### 2.1. Participants

Twenty patients (eight males, all right-handed, mean age of 75.95 years; range, 55–85 years) with diagnosed age-related macular degeneration participated after providing informed consent. The study was approved by the local ethics review board of the Medical Faculty of Otto-von-Guericke University (OVGU) (Nr. 76/08 of 14.06.2012 and 72/18 of 03.07.2018). Twelve patients were recruited and diagnosed at the Ophthalmic Department at the University Hospitals of the Otto-von-Guericke University, Magdeburg (OVGU), and eight patients were recruited and diagnosed at the Eye Center am Johannisplatz (ECL) in Leipzig. Macular degeneration was determined based on an ophthalmic examination provided at the eye clinics. At ECL, the presence of absolute scotomas was monitored by using the Amsler grid, and at OVGU, standard perimetry (OCTOPUS Perimeter 101 (dynamic strategy; Goldmann size III; program: dG2; Haag-Streit GmbH, Wedel, Germany) or MP1 (Nideck Micro-Perimeter)) was applied identify and discriminate between relative and absolute scotomas. With one exception, all patients were affected by AMD in both eyes, but the severity of the pathology varied across subjects (Table 1). Subjects with narrow iridocorneal angle, glaucoma, ocular trauma, eye surgery, (except cataract surgery), advanced cataract, diabetic retinopathy and other retinal diseases, high myopia (>5 dpt), amblyopia, cerebral blood flow disorder, and stroke were excluded (see Table 1). Fourteen patients performed the experiment with monocular and 6 patients with binocular vision. From the 14 monocularly tested patients, 9 were tested with their worse eye and 5 with their better eye, because testing with the worse eye was not possible.

In addition to the patients, we tested twenty controls binocularly (four males, all right-handed, mean age of 72.45 years; range, 66–79 years). Testing took place at Otto-von-Guericke University, Magdeburg. Controls’ acuity was examined using the Freiburger Vision Test 3.9.9. [17]. All controls had normal or corrected to normal visual acuity (decimal visual acuity > 0.84).

Participants remained naive to the purpose of the research during the experiments. None of them had been tested in a similar study before. Immediately after the experiment, individuals from both groups received a fixed reimbursement of EUR 20 for the one-hour session.

### 2.2. Stimuli

**Figure 1:**
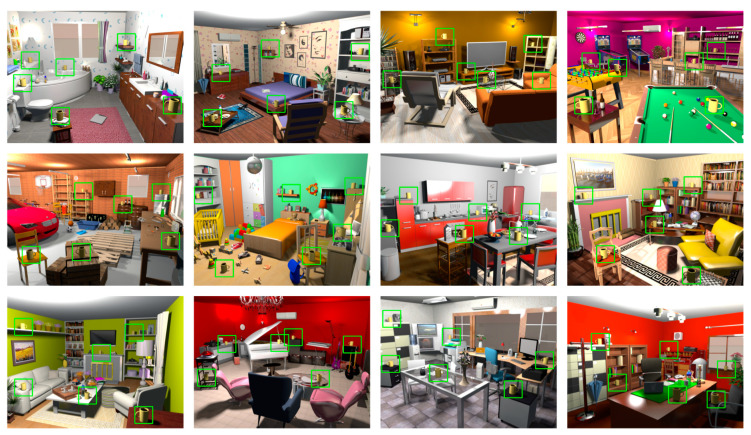
Interior scenes used in the experiment. The task was to search for the yellow mug and to indicate the left/right direction of the handle by an alternative forced choice response. For each scene, all six possible targets and their locations are shown. In the experiment, however, each scene contained only one target. In repeated displays, the target occurred at the same location across repetitions whereas in new displays, the target appeared once at each of the shown positions across repetitions. The green squares indicating target locations are for illustrative purposes and were not presented during the experiment.

The stimuli were presented and recorded by a Lenovo ThinkPad L420 laptop (Linux operating system, version 8) using PsychoPy v1.82.01 Software [18] under Python. The laptop was connected to a full-color 24-inch HD LCD BenQ XL2410T presentation monitor. The monitor was 521-mm (1920 pixels) wide and 293-mm (1080 pixels) high, with a refresh rate of 120 Hz. Stimuli were presented at a distance of 80 cm in a quiet and dimly-lit room. The stimuli were designed with Sweet Home 3D software [19]. Twelve 3D-rendered illustrations of naturalistic scenes of 22.8° × 17.1° visual angle represented a bathroom, bedroom, cinema room, game room, garage, nursery room, kitchen, library, living room, music room, office, and studio (Figure 1).

A yellow mug with a handle pointing either left or right constituted the visual search target and was presented in one of six equal-sized rectangular parts of the display (upper/lower, left/center/right). Each display contained only one mug. The mug could appear both in familiar positions as well as in unfamiliar positions in the rooms, but not in physically impossible places, e.g., up in the air (Figure 1). Depending on the position, the size of the mug varied between ca. 1.1° × 1.1° and 0.6° × 0.6°. The orientation of the handle (left or right) was chosen randomly and balanced in each block.

### 2.3. Procedures

A trial began with the presentation of a central black fixation cross with a line length of 2.5° for 1000 ms. After a blank screen of 500 ms, the display was presented until participants indicated the direction of the mugs handle by an alternative button press response. After another blank screen for 500 ms, the next trial began. Participants had to indicate the direction of the mug’s handle by pressing the left or right mouse button. A 2000-Hz high-pitch tone provided positive auditory feedback and a 500-Hz low-pitch tone provided negative auditory feedback.

**Figure 2:**
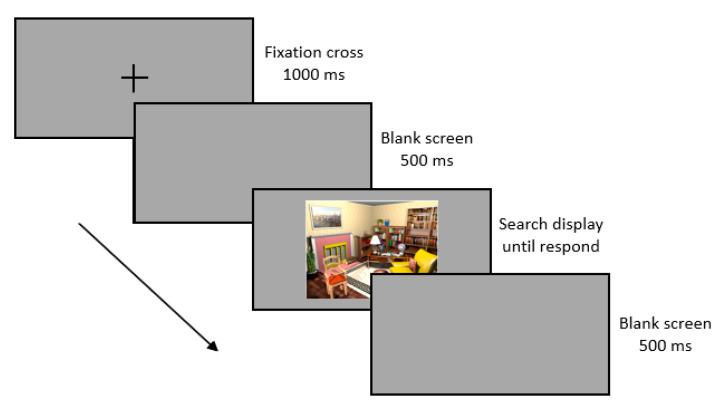
**Top**: Example trial.

The visual search task consisted of six blocks, with each block including 12 trials, and each trial with a different room as search display. These 12 rooms were repeated in each block in a random order. In six of the 12 rooms—the repeated displays—randomly drawn for each participant, the mug’s position was randomly drawn for each individual for the first block and then fixed across repetitions (blocks), whereas in the other six displays—the new displays—the mug’s position varied randomly across the six rectangular sections, with the restriction that no location was used twice in a room. In this way, the probability of target presentation was about the same across the six rectangular areas of a grid created by a horizontal line through the center of the scene and two vertical lines dividing the scene into equally wide sections), both for new and repeated locations, thus preventing a confound of target location cueing and contextual cueing. Observers were not informed about target location repetitions or scene repetitions. Figure 2 shows an example of a trial.

### 2.4. Recognition Test

Subsequent to the experiment, we ran an explicit recognition test in order to assess whether the repeated scenes were explicitly or implicitly remembered. The six previously repeated scene configurations were presented without the search target (mug). The participant’s task was to indicate the target’s position for each specific scene by pointing with the mouse cursor. Recognition accuracy was operationalized as the frequency of correct mouse cursor placements in the target display section of the 2 × 3 grid (see last paragraph).

### 2.5. Data Analysis

All trials with incorrect responses (3% for patients; 1% for controls) and trials in which the response time exceeded the outlier threshold of ± 2 standard deviations from the mean (patients: 2.7% of repeated, 3.3% of new trials; controls: 2.4% in both trials groups) were excluded from the response-time analyses. Mean response times (RTs) were determined separately for repeated and new configurations for each subject and block. To increase statistical power, RTs were aggregated to three epochs, with each epoch containing two subsequent blocks. Next to RTs, because of the different overall response times between patients and controls, normalized contextual cueing effects [(RT(new) − RT(repeated))/RT(new)] were analyzed. Thus, positive values indicate a benefit for repeated configurations. When Mauchly’s test indicated that the assumption of sphericity had been violated, degrees of freedom were corrected using Greenhouse–Geisser estimates of sphericity. For all statistical tests, the alpha level was set at 0.05.

## 3. Results

### 3.1. Response Times

We first investigated potential effects of monocular vs. binocular viewing on RT in the patients. A repeated measures ANOVA with group (monocular, binocular) as the between-subjects factor and configuration (repeated, new) and epoch (1–3) as within-subjects factors yielded neither a significant effect of group (*F*(1, 18) = 0.133, *p* = 0.72, *η_p_*^2^ = 0.007) nor significant interactions with group (configuration × group: *F*(1, 18) = 0.021, *p* = 0.886, *η_p_*^2^ < 0.001; epoch × group: *F*(2, 36) = 0.648, *p* = 0.53, *η_p_*^2^ = 0.035; configuration × epoch × group: *F*(2, 36) = 1.218, *p* = 0.31, *η_p_*^2^ = 0.063). Because of the absence of ocularity effects on visual search, we collapsed over ocularity in all further analyses to increase power.

**Figure 3:**
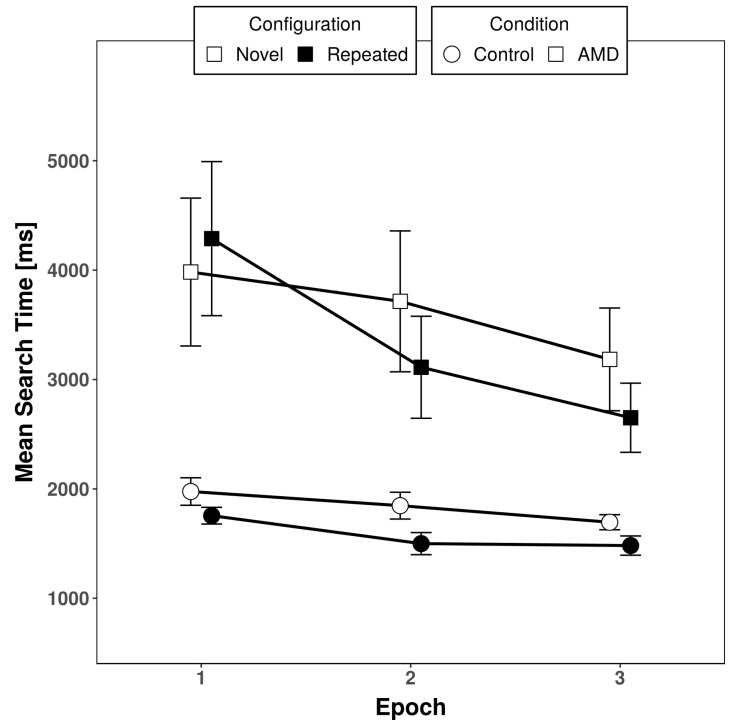
Averaged response times for controls (circles) and age-related macular degeneration (AMD) patients (squares) as a function of repeated (filled symbols) and novel (open symbols) search displays. Error bars depict the standard error of the mean.

Averaged response times for patients and controls are shown in Figure 3 and Table 2. A repeated measures ANOVA with group (patients, controls) as the between-subjects factor and configuration (repeated, new) and epoch (1–3) as within-subjects factors yielded a significant main effect of group (*F*(1, 38) = 11.410, *p* = 0.002, *η_p_*^2^ = 0.231), yielded by overall faster response times of controls (1709 ms) compared to patients (3489 ms). The main effect of configuration was also significant (*F*(1, 38) = 7.361, *p* = 0.01, *η_p_*^2^ = 0.162), indicating overall shorter response times in repeated displays (2465 ms) compared with novel displays (2733 ms), as expected due to contextual cueing. The significant main effect of epoch (*F*(2, 76) = 14.542, *p* < 0.001, *η_p_*^2^ = 0.277) reflected decreasing response times over epochs, indicating general learning (3001 ms in epoch 1 to 2253 ms in epoch 3). Importantly, the interaction between epoch and configuration was significant (*F*(2, 76) = 3.547, *p* = 0.034, *η_p_*^2^ = 0.085), indicating the development of contextual cueing over time (difference in novel/repeated display RT of −42.4 ms in the first epoch compared to 475 and 374 ms in the second and third epochs, respectively). Epoch interacted significantly with group (*F*(2, 76) = 5.696, *p* = 0.005, *η_p_*^2^ = 0.130). Patients showed, overall, a much stronger decrease in response times over epochs than controls (1218-ms improvement from the first to third epoch in patients vs. 277-ms improvement in controls). The remaining interactions including the factor group were not significant, indicating that contextual cueing was comparable between patients and controls (configuration × group: *F*(1, 38) = 0.007, *p* = 0.934, *η_p_*^2^ < 0.001; configuration × epoch × group: *F*(1, 76) = 2.620, *p* = 0.110, *η_p_*^2^ = 0.065).

Separate repeated measures ANOVAs for each group with the within-subjects factors configuration (repeated, new) and epoch (1–3) were performed on response times to ensure that contextual cueing was reliable in patients as well as controls.

In controls, the significant main effect of epoch indicated general learning (*F*(2, 38) = 14.665, *p* < 0.001, *η_p_*^2^ = 0.956) and the significant main effect of configuration revealed contextual cueing (*F*(1, 19) = 20.916 *p* < 0.001, *η_p_*^2^ = 0.524). The interaction was not significant (*F*(2, 38) = 0.932, *p* = 0.403, *η_p_*^2^ = 0.047), indicating that strong contextual cueing effects developed from early on.

In patients, the significant main effect of epoch indicated general learning (*F*(2, 38) = 9.953, *p* < 0.001, *η_p_*^2^ = 0.344). The main effect of configuration was not significant (*F*(1, 19) = 2.131 *p* = 0.161, *η_p_*^2^ = 0.101), but the significant interaction (*F*(2, 38) = 3.249, *p* = 0.0498, *η_p_*^2^ = 0.146) confirmed that contextual cueing was building up over time in the patient sample (Figure 3). This was confirmed by a paired samples *t*-test on the collapsed epochs 2 and 3 which yielded significantly faster RTs for repeated displays (*t*(19) = 2.449, *p* = 0.024, d = 0.548).

The ANOVA hinted at a later onset of contextual cueing in the patient sample. We therefore calculated paired samples *t*-tests over the collapsed epochs 2 and 3, separately, for patients and controls. Both patients (*t*(37) = 2.19, *p* = 0.035) and controls (*t*(39) = 4.898, *p* < 0.001) showed a significant response-time advantage for repeated displays.

To investigate the evidence for equal contextual cueing strength in both groups [20], we calculated a Bayes factor analysis on contextual cueing scores in epochs 2 and 3. Using Jasp [21], a Bayesian *t*-test for independent samples compared the fit of the data under the null hypothesis of no difference and the alternative hypothesis of different cueing effects between the control group and AMD patients. The analysis yielded a BF_01_ = 1.851, i.e., only weak evidence for equality of contextual cueing effects in the two groups.

Normalized contextual cueing scores were analyzed because of the overall longer response times of the patients with a repeated measures ANOVA with the within-subject factor epoch (1–3) and the between-subjects factor experimental group (patients, controls). Standardized cueing scores did not differ significantly between groups (*F*(1, 38) = 2.656, *p* = 0.111, *η_p_*^2^ = 0.065). Moreover, neither the main effect of epoch (*F_GG_*(1.521, 57.817) = 0.948, *p* = 0.371, *η_p_*^2^ = 0.024) nor the group × epoch interaction (*F*(2, 76) = 0.748, *p* = 0.477, *η_p_*^2^ = 0.019) were significant. Thus, relative to the baseline differences, patients did not reduce their response times more than controls in the course of the experiment. Moreover, there was, again, no indication of a contextual cueing impairment in the patients.

An analogous Bayesian *t*-test for the RT yielded a BF_01_ = 2.148, indicating, again, only weak evidence for equal size of contextual cueing in patients and controls [22].

**Figure 4:**
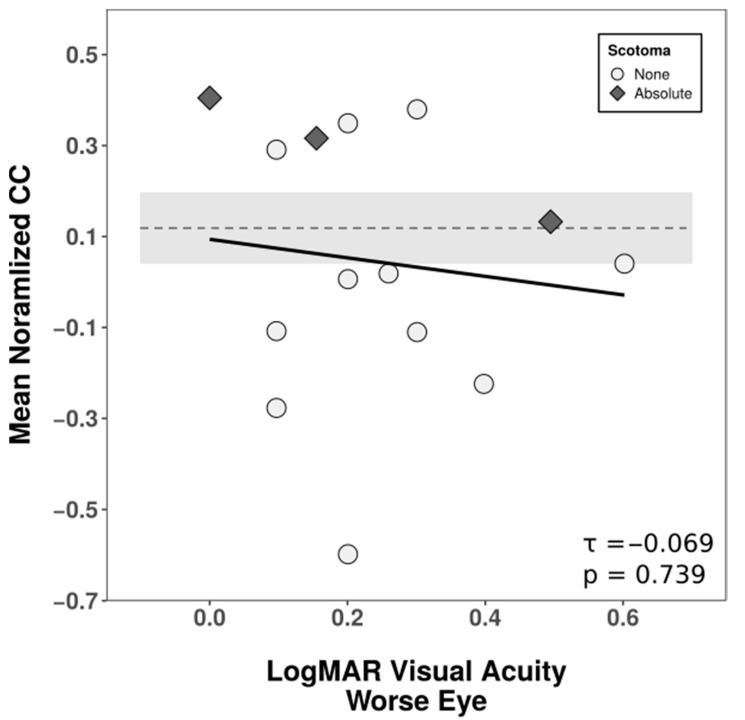
Relationship between the degree of foveal impairment in AMD patients (logMAR visual acuity worse eye) and normalized contextual cueing in response time in the last epoch in patients tested monocularly. Normalized contextual cueing was obtained by individually calculating the difference in mean response times between novel and repeated displays and standardizing this absolute difference by the mean response time of novel displays. Positive values indicate a benefit for repeated configurations. Rank correlations were quantified using Kendall’s τ. The solid line depicts the linear regression for the purpose of visualization. The dashed line represents the averaged mean normalized contextual cueing of controls, and the shaded area, the corresponding 95% confidence intervals.

If contextual cueing is preserved in AMD patients, when searching in real world scenes, contextual cueing scores should, overall, be unrelated to the degree of foveal vision impairment. To test this prediction, we correlated logMAR visual acuity as a measure of general visual performance and the normalized contextual cueing effect in repeated displays in the last epoch using Kendall’s τ non-parametric rank order correlation (Figure 4). Only patients who had performed the experiment monocularly were considered for this analysis (*n* = 14). LogMAR visual acuity of the tested eye did not correlate significantly with the size of contextual cueing (τ = −0.069, *p* = 0.739), implying that contextual cueing was also reliable in more severely impaired patients.

### 3.2. Accuracy

Response time is the central variable of interest in the contextual cueing paradigm [23] and accuracy is usually high in contextual cueing paradigms because participants have no time limit. This was also observed in the present study, both for patients (monocular: mean 96%; binocular: mean 97%) and controls (mean: 99%). Thus, whereas reaction times are the main variable of interest, accuracy was, nevertheless, analyzed to rule out potential speed–accuracy trade-offs.

A repeated measures ANOVA comparing accuracy with the between-subjects factor experimental group (control group, AMD monocular, AMD binocular) and the within-subjects factors configuration (novel, repeated) and epoch (1–3) revealed no significant difference between groups (*F*(2, 37) = 2.350, *p* = 0.109, *η_p_*^2^ = 0.113). The only significant effect was the main effect of epoch (*F*(2, 74) = 3.530, *p* = 0.034, *η_p_*^2^ = 0.087) due to a slight improvement in accuracy after the first epoch (epoch 1, 97%; epoch 2, 99%; epoch 3, 98%). No other effect approached significance (all Fs < 1.944, *p* > 0.111, *η_p_*^2^ < 0.095).

### 3.3. Recognition Test

The experiment was followed by an explicit recognition test to address the question of whether learning was implicit or explicit. For this purpose, the displays with repeated target locations were presented again and the participants had to indicate the target sector (see Methods). The average recognition accuracy in patients was 39.16%. To test whether the participants were able to recall the position of the target, we compared the accuracy obtained in the sample with the chance level of 16.6%, since in each trial, the probability to select the correct position was 1/6. Patients’ accuracy was significantly above the chance level (*t*(19) = 5.107, *p* < 0.001), indicating that they recalled the repeated target locations. For the controls, accuracy was 58.33%. This, too, was significantly above the chance level (*t*(19) = 7.611, *p* < 0.001). Recognition accuracy was significantly increased for controls compared with patients (*t*(19.167) = 2.728, *p* ≤ 0.01).

## 4. Discussion

We investigated contextual cueing of visual search in repeated scenes in patients with AMD. In previous studies with patients with AMD [7] as well as simulated central vision loss in normal-sighted observers [8,9], impairment of contextual cueing was observed during search with foveal vision loss. In these studies, artificial displays were presented in which a T-shaped target had to be searched among L-shaped distractors. In the present study, we investigated if contextual cueing was preserved in patients with AMD when searching in real-world scenes. In line with previous work [1], we found faster response times in displays with repeated target locations, indicating contextual cueing.

Although this search facilitation was numerically somewhat smaller in the patients than the controls, we observed no significant group × configuration interaction, which would have been indicative of group differences in the amount of contextual cueing. Nevertheless, we cannot make a firm conclusion about the equality of the size of the contextual cueing effects in AMD patients and controls, as the Bayes factor analyses yielded only evidence for a roughly two-fold higher probability for equal than for unequal size. The important point, however, is that the patients showed significantly faster search times in repeated displays after the first third of the experiment, indicative of contextual cueing.

Visual search in general—for new and repeated target locations alike—was much slower in the patient group, as was expected, due to their impaired vision. While contextual cueing manifested itself in the faster search for targets at constant target locations, there was also an unspecific learning effect that led to faster search for both constant and variable target locations from epoch to epoch. This general learning effect was present in patients and controls alike. It may even have been somewhat stronger in the patients, as suggested by the group × epoch interaction in the age-matched patient sample (and the trend in the overall patient sample). This effect, however, could be attributed to their higher initial response times.

We confirmed the central hypothesis of this paper that the clear contextual cueing deficit that we observed in previous work with AMD patients [7] would be ameliorated for search in realistic displays. Our reasoning was that realistic displays would enable explicit memory of the target location in the form of a semantic template (e.g., the cup is on the kitchen table). In turn, this would resolve the competition between concurrent visuospatial working memory demands for top-down controlled exploration of the scene (e.g., navigating the fixation to the table), on the one hand, and keeping a detailed visuospatial memory template active during search. In contrast, in symbolic displays, visuospatial working memory is needed both for keeping the contextual memory template active during search for comparison with the display and to navigate the display with eye movements concurrently [9]. In keeping with this reasoning, we observed that patients and controls alike could explicitly remember the repeated target locations, unlike the AMD patients tested with symbolic displays in our previous contextual cueing study [7]. Moreover, visual scene exploration was clearly impaired in the patients, indicated by their much longer response times. Thus, visuospatial working memory demands most likely were as high as, if not higher than, the patients with AMD of our previous study [7]. This supports our view that patients did not use visuospatial working memory but semantic memory to guide visual search in the present experiment.

The fact that contextual cueing in naturalistic scenes went along with explicit memory in our study—in keeping with previous work with normal-sighted observers—does not imply that object or scene recognition has to occur explicitly in AMD. In fact, scene categories can be distinguished after brief presentation in the peripheral visual field [24] and scenes may implicitly prime recognition of congruent objects in AMD patients [25]. In the present displays with repeated target location, the scene category (e.g., kitchen or living room) may have been rapidly identified, leading to the memory trace linking the particular scene to the associated target location.

Although this study was inspired by our previous work on contextual cueing in AMD patients, a direct comparison is limited by the fact that different patient samples were tested. Thus, the present results should be seen as evidence that contextual cueing in realistic displays is possible in AMD patients, not as a direct comparison of contextual cueing strengths between these studies. A direct comparison of contextual cueing in symbolic and realistic displays would further by complicated by the inherent differences of naturalistic and symbolic displays. It is, perhaps, noteworthy that we reported a search advantage for AMD patients in naturalistic displays which was not observed in symbolic displays, whereas one could assume that the greater visual complexity of naturalistic displays might cause problems for patients with visual deficits, as has been reported in a study of glaucoma patients [26]. A worthwhile issue for future studies would be to compare search in familiar scenes (as in the present study) with search in unfamiliar scenes. A disadvantage for the latter might support our interpretation that the semantic content of the scene helps to reduce working memory load during contextual search guidance.

In a recently published study, we carried out the same experiment with the same scenes as in the present study in healthy younger observers under scotoma simulation [16]. Regarding contextual cueing, improvement of exploration efficiency was very similar to unimpaired observers in our previous work using arbitrary search displays [7,8,9] in that fixation number was significantly reduced and scan paths showed a tendency towards increased efficiency in repeated compared to novel displays. Furthermore, the onset of the monotonic path that distinguishes an early inefficient search phase from the efficient guidance of the eye to the target with each successive fixation occurred significantly earlier in repeated than novel displays. Interestingly, we found comparable contextual cueing effects between search with or without a simulated scotoma using the realistic scenes [16], suggesting that even observers without previous vision-loss experience may better profit from contextual cues to guide their eye movements in realistic scenes than in arbitrary search displays. This was the case, although exploration was generally more complicated, reflected by more fixations that were longer in duration and increased saccade amplitudes. Thus, future work may want to investigate eye movements—perhaps in parallel in AMD patients and normal-sighted participants with gaze-contingent simulated scotomata—in order to see if patients show comparable eye-movement patterns—e.g., less fixations of longer duration and higher saccade amplitudes—as can be induced by simulated central scotomata [9,27].

Unfortunately, it was not possible to perform eye tracking in the present study. In a recently published study, however, we carried out the same experiment using realistic scenes in healthy younger observers with scotoma simulation [16]. Regarding contextual cueing, improvement of exploration efficiency was very similar to unimpaired observers in our previous work using arbitrary search displays [7,8,9] in that fixation number was significantly reduced and scan paths showed a tendency towards increased efficiency in repeated compared to novel displays. Furthermore, the onset of the monotonic path that distinguishes an early inefficient search phase from the efficient guidance of the eye to the target with each successive fixation occurred significantly earlier in repeated displays than novel displays.

Beyond the issue of contextual search guidance, the present data show that the use of realistic stimuli may contribute to the question of how well laboratory experiments can predict behavior and its limitations due to pathology in real life. The contextual cueing deficits that we observed in our previous work in AMD patients [7] may be compensated for in everyday situations by the use of semantic memory templates. The size of the search advantage due to contextual cueing was in the order of several hundred ms in the present study, rendering it a factor that should be of importance for visual search in everyday situations.

## Figures and Tables

**Table 1:** Patient characteristics. Notes: RE, right eye; LE, left eye; AMD, age-related macular degeneration; –, no scotoma; r, relative scotoma; a, absolute scotoma; ^ag^, tested with Amsler grid; * tested eye. Patients S1–S6 were tested binocularly.

			Diagnoses		Acuity		Scotoma	
Subject	Sex	Age (years)	RE	LE	RE	LE	RE	LE
S01	F	61	AMD(wet)	AMD (dry)	0.6	1.0	r	r
S02	F	80	AMD(wet)	/	0.1	0.8	r	r
S03	F	78	AMD(wet)	AMD (wet)	0.7	1.0	r	r
S04	F	79	AMD(wet)	AMD (wet)	0.3	0.05	r	r
S05	F	76	AMD(wet)	AMD (wet)	0.7	0.7	r	r
S06	F	55	AMD(wet)	AMD (wet)	0.8	0.8	r	r
S07	M	81	AMD (dry)	AMD (dry)	0.8	0.5/0.6	r	r*
S08	M	74	AMD (dry)	AMD (dry)	0.04	0.5	a	r*
S09	F	80	AMD (dry)	AMD (dry)	0.5	0.32	–^ag^	–^ag^*
S10	F	84	AMD (dry)	AMD (dry)	1.0	0.8	–^ag^	–^ag^*
S11	M	71	AMD (dry)	AMD (dry)	0.63	0.63	–^ag^	–^ag^*
S12	F	78	AMD (dry)	AMD (wet)	0.8	0.4	–^ag^*	–^ag^
S13	M	80	AMD (dry)	AMD (dry)	0.63	0.63	–^ag^*	–^ag^
S14	F	75	AMD (dry)	AMD (dry)	0.03	0.8	a^ag^	–^ag^*
S15	M	79	AMD (dry)	AMD (dry)	0.63	0.63	–^ag^	–^ag^*
S16	F	79	AMD (dry)	AMD (dry)	0.8	0.8	–^ag^*	–^ag^
S17	M	80	AMD (dry)	AMD (wet)	0.32	0.08	a*	a
S18	M	76	AMD (dry)	AMD (dry)	1.0	0.25	–*	r
S19	F	72	AMD (dry)	AMD (dry)	0.7	1.0	a*	r
S20	M	80	AMD (dry)	AMD (wet)	1.0	0.08	r*	a

**Table 2:** Mean difference between novel and repeated configurations and normalized contextual cueing effects ((RTnovel − RTrepeated)/RTnovel).

	Epoch 1		Epoch 2		Epoch 3	
Condition	Absolute Mean (SD)	Normalized Mean (SD)	Absolute Mean (SD)	Normalized Mean (SD)	Absolute Mean (SD)	Normalized Mean (SD)
Control	221 (421)	0.07 (0.18)	347 (420)	0.14 (0.17)	213 (288)	0.12 (0.17)
Patient	−306 (1281)	−0.17 (0.35)	603 (1470)	0.07 (0.25)	534 (1227)	0.08 (0.28)
